# 

**Published:** 1877-11

**Authors:** 


### Contents of November Number.

### ORIGINAL.

ART. I.—Surgical Reports. By Charles A. Wall, B. S. Cases taken from the Practice of Prof. J. F. Miner, M. D. Incised wound of knee and division of biceps.—Compound fracture of leg. Recovery.—Compound fracture. Amputation.—Psoas abcess. 121
ART. II.—Pyo-Pneumo-Thorax. By Prof. E. V. Stoddard, M. D. 131
ART. III.—Proceedings of the Buffalo Medical Association,........ 133

### MISCELLANEOUS.

Antiseptic Surgery. A. C. Girard, M. D.,........................ 137
Bordeaux method of Dressing Wounds,........................ 145
A Growing Nuisance,........................................ 146
OBITUARY.
Death of Prof. George Hadley, M. D.,.................... 146

### EDITORIAL.

Charities of Physicians,—Hospitals,—Free Dispensaries, etc.,—Their Influence upon the profession and public,................ 150
Newspaper Law Decisions,........................................ 153
BOOKS REVIEWED:
Transactions of American Medical Association. Prize Essay,........................................ 153
Ziemssen's Cyclopœdia. Vol. XV,........................ 154
Transactions of the College of Physicians of Philadelphia,.. 157
Transactions of the Kentucky State Medical Society,........ 157
The Microscope. John Phin,.............................. 158
Hospitals. W. Gill Wylie, M. D.,........................ 158
Diseases of the Skin. Louis A. Duhring, M. D.,........... 159
Surgical Observations. J. Mason Warren, M. D.,........... 160
Books and Pamphlets Received,.............................. 160

